# Endoscopic antireflux mucoplasty using the reopenable-clip over-the-line method for refractory gastro-esophageal reflux disease

**DOI:** 10.1055/a-2719-8037

**Published:** 2025-11-19

**Authors:** Sayuri Hashimoto, Satoshi Asai, Tomoya Hashimura, Kotaro Takeshita, Yuki Kano, Eisuke Nakao, Eisuke Akamine

**Affiliations:** 1 38434 Department of Gastroenterology, Tane General Hospital, Osaka, Japan

Endoscopic antireflux mucoplasty (ARMP) has been proposed to overcome the limitations of antireflux mucosectomy 1 , such as delayed bleeding and unpredictable ulcer scar contraction, by promoting ulcer closure 2 . However, achieving reliable closure of the cardiac ulcers during ARMP remains technically challenging. The reopenable-clip over-the-line method (ROLM) – an ulcer closure technique using endoscopic clips and nylon thread – has been previously described 3 . Based on these findings, our institution adopted ARMP using the ROLM. A 54-year-old man presented with a 10-year history of a burning throat sensation caused by refractory gastro-esophageal reflux disease (GERD). Esophagogastroduodenoscopy (EGD) revealed a hiatal hernia (CO-SH scale 4 : CO2-SH1, Hill grade 2) ( Fig. 1 **a, b** ). Symptom scores at presentation were as follows: GERD-HRQL 13, GerdQ 3, and FSSG 14. Despite antacid therapy, 24-h pH monitoring revealed 50 reflux episodes. The patient subsequently underwent ARMP for refractory GERD ( Video 1 ). Endoscopic aspiration mucosectomy was performed semicircumferentially, just below the squamocolumnar junction ( Fig. 1 **c** ). The resulting mucosal defect was closed using ROLM with 14 reopenable clips. Tightening of the esophagogastric junction was confirmed using a thin endoscope immediately after the procedure ( Fig. 1 **d** ). The procedure duration was 30 min for mucosal resection and 35 min for closure with no complications. Oral intake was initiated on postoperative day 2, and the patient was discharged on day 3. At the 3-month follow-up, symptom scores had significantly improved (GERD-HRQL, 7; GerdQ, 0; and FSSG, 3). EGD showed improvement in the hiatal hernia to CO1.1-SH0 and Hill grade 1 ( Fig. 1 **e** ). Antacid therapy was no longer necessary. These findings demonstrate that ROLM is a simple and effective technique for ARMP.

**Fig. 1:**
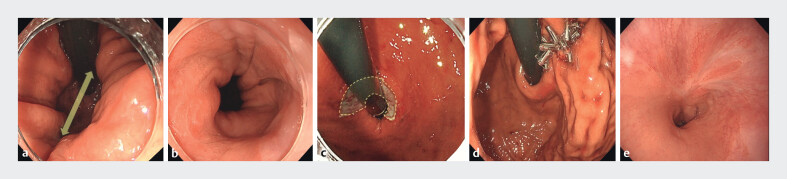
**a, b** Esophagogastroduodenoscopy (EGD) prior to antireflux mucoplasty (ARMP) showing a hiatal hernia (CO-SH scale: CO2-SH1, Hill’s grade 2). **c** Endoscopic aspiration mucosectomy was performed at four sites on the gastric mucosa just below the squamocolumnar junction, resulting in a semicircular mucosal resection. **d** The ulcer was then closed using nylon thread and endoscopic clips via the reopenable-clip over-the-line method. **e** Post-ARMP EGD shows ulcer scar contraction and narrowing of the esophagogastric junction.

Endoscopic antireflux mucoplasty for refractory gastro-esophageal reflux disease with closure of the mucosal defect using the reopenable-clip over-the-line method.Video 1

Endoscopy_UCTN_Code_TTT_1AO_2AJ

## Notes

Endoscopy E-Videos https://eref.thieme.de/e-videos
*E-Videos* is an open access online section of the journal *Endoscopy* , reporting on interesting cases and new techniques in gastroenterological endoscopy. All papers include a high-quality video and are published with a Creative Commons CC-BY license. Endoscopy E-Videos qualify for HINARI discounts and waivers and eligibility is automatically checked during the submission process. We grant 100% waivers to articles whose corresponding authors are based in Group A countries and 50% waivers to those who are based in Group B countries as classified by Research4Life (see: https://www.research4life.org/access/eligibility/ ). This section has its own submission website at https://mc.manuscriptcentral.com/e-videos .
